# Modulus of Elasticity of Two Ceramic Materials and Stress-Inducing Mechanical Deformation following Fabrication Techniques and Adhesive Cementation Procedures of a Dental Ceramic

**DOI:** 10.1155/2019/4325845

**Published:** 2019-11-19

**Authors:** G. Isgrò, D. Rodi, A. Sachs, M. Hashimoto

**Affiliations:** ^1^CAD-CAM Dental Technology Centre, Via Del Mare 3, 98051 Barcellona Pozzo di Gotto, Italy; ^2^Consultant, Ferrara, Italy; ^3^Consultant, Amsterdam, Netherlands; ^4^Faculty of Health Sciences, Department of Oral Health Sciences, Osaka Dental University, 1-4-4 Makinohonmachi, Hirakata, Osaka 573-1144, Japan

## Abstract

**Statement of Problem:**

Fabrication technique, precementation, and cementation operative procedures can induce significant modification of the stressing patterns throughout the thickness of some classes of dental ceramic materials.

**Objectives:**

To estimate, by means of the deflection test, residual stress in restorative dental ceramic following fabrication technique, precementation, and resin cement coating procedures and to relate it to the elastic property of the ceramic material tested.

**Materials and Methods:**

From IPS e.max® Press, lithium disilicate heat-pressed glass-ceramic (elastic modulus of 95 ± 5 GPa) disc-shaped specimens (*n* = 10) were made according to the manufacturer's instructions. One surface of the specimens was polished to provide accurate baseline profilometric measurements (reference surface). Deflection measurements were performed after polishing and annealing alumina air-particle abrasion of the unpolished surface followed by resin cement coating of the alumina air-particle abraded surface. The specimens were reprofiled at 24, 48, and 168 hrs after coating. The Friedman test followed by Dunn's multiple comparison test was employed to identify significant differences (*p* < 0.05). To compare the difference in mean of maximum mechanical deflection, after cement coating at 0 hr, between two different ceramic materials (IPS e.max Press and Vitadur Alpha (result from another study)), Student's *t*-test for unpaired data was performed.

**Results:**

Baseline profilometric measurements identified a convex form on the polished surface of the ceramic discs with a mean of maximum mechanical deflection of 4.45 ± 0.87 *μ*m. A significant reduction in convexity of the polished specimens was characterized after alumina air-particle abrasion of the unpolished surface. The mean deflection significantly increased after resin cement coating and did not change over the time investigated.

**Conclusions:**

The precementation treatment, namely, alumina air-particle abrasion and cementation procedure of IPS e.max® Press glass-ceramic disc-shaped specimens generates stress that induced mechanical deformation. However, a dental ceramic material with higher elastic modulus (stiffer) would minimize stress-inducing mechanical deformation.

## 1. Introduction

Aesthetic dental ceramic materials have been used to manufacture porcelain laminate veneer (PLV) and dentine-bonded crown (DBC) dental restorations. However, the fracture strength of a dental ceramic as for a glass is dependent to surface defects which generate cracks that propagate, under tensile stress, through the bulk of the material [1]. Fractographic analysis of clinically failed ceramic crowns [2] and finite-element analysis [3] employed to identify stress distribution in ceramic crown have reported the development, under occlusal load, of high tensile stress on the fit surface of the restorations. Therefore, the quality of the surface finishing of a dental ceramic has an effect, during masticatory functions, on the longevity of the restoration.

In the last decade, ceramic restorations have received a considerable interest by the clinicians owing to the improvement in fracture resistance of the ceramic material. The enhanced fracture resistance of the dental ceramic has been attributed to the increasing amount of crystals in the glass matrix (i.e., dispersion strengthening) to impede the propagation of crack [4] and to the heat-pressing technique that reduces the formation, in the microstructure, of voids and allows a homogenous distribution of the crystals in the glass matrix [5, 6]. Resin-based cements used to lute the restoration to the tooth structure have also increased the resistance to fracture of a dental ceramic [7, 8]. The mechanism that accounts for the increase in strength is associated to the infiltration by the resin cement of defects present on the fit surface of the ceramic during the coating procedure forming, thereby, a resin-ceramic hybrid layer [9]. The volumetric shrinkage, of the resin cement within the hybrid layer following polymerization, generates a state of compression stress that induces the closure of cracks [10].

Based on the dispersion strengthening method, dental ceramic materials with new chemical composition have been developed for fabrication, by means of heat-pressing forming techniques, of ceramic restorations that have a flexural strength ranging from 160 to 350 MPa [11, 12]. Ivoclar-Vivadent has introduced a heat-pressed glass-ceramic named IPS e.max® Press with a microstructure that consists of 70 vol % of lithium disilicate crystals embedded in a glass matrix which is basically a further development of IPS Empress 2. The biaxial flexural strength (BFS) and elastic modulus are reported by the manufacturer to be 440 ± 55 MPa and 95 ± 5 GPa, respectively [13]. The IPS e.max® Press glass-ceramic material is indicated for the fabrication of veneers, owing to the good optical properties, single crowns, and three-unit bridge dental ceramic restorations placed in the anterior region of the mouth [13].

The strength of a dental ceramic is not only controlled by the mechanical property of the material and/or surface finishing of the restoration but also by residual stress induced in the ceramic restoration as a result of firing procedures (i.e., cooling from furnace temperature to room temperature) to manufacture the restoration [14, 15], precementation treatment of the fit surface, and cementation procedure [16].

Isgrò and coauthors have introduced a method, “the deflection test” [15, 16], that can be used to characterize, in disc-shaped ceramic specimen, residual stress state with a magnitude lower than that required for crack propagation. A ceramic disc that is free of stress is expected to be flat. However, if the profile of the ceramic disc characterized, by profilometric measurements, is convex, it represents an error from the expected flatness and indicates that a surface is in a state of tensile stress and the opposite surface of the ceramic disc is in a compression stress state [17]. From the amplitude of the convexity, the mean of maximum mechanical deflection is calculated to estimate residual stress. However, the deflection test requires accurate profilometric measurements; therefore, one surface of the ceramic disc is polished to reduce the surface defects (<4 *μ*m) and to provide a baseline reference deflection value.

The deflection test has been previously employed by the author to characterize thermal stress-inducing mechanical deformation of IPS Empress 2 heat-pressed ceramic core material following the application of veneering porcelains with a range of thermal contraction coefficient mismatch values [15]. In another study [16], for Vitadur Alpha (alumina filled (10% vol) feldsphatic porcelain material with an elastic modulus of 65 GPa), the authors were able to estimate the magnitude of induced stress in ceramic discs following alumina air-particle abrasion treatment (precementation technique) and resin cement coating procedure. The result of the study suggested that alumina air-particle abrasion treatment induced tensile stress, thereby decreasing the strength of Vitadur Alpha ceramic material [18], whereas compression stress set by the volumetric shrinkage of the resin cement induced mechanical deformation of the ceramic disc throughout its thickness.

The aims of the investigation were to characterize, with the deflection test, residual stresses in IPS e.max® Press glass-ceramic discs following preparation of the specimens and how these stresses modify as a result of alumina air-particle abrasion treatment and resin cement coating procedures. Furthermore, the result from this study was also compared with the result previously obtained for Vitadur Alpha [16] to relate the elastic properties of the ceramic materials tested and the effect of the resin cement coating procedure. The hypotheses to be tested were as follows: first, fabrication techniques (i.e., sintering or heat pressing) might introduce stress that could produce mechanical deflection in a dental ceramic and second, a ceramic material with high elastic property would minimize stress-inducing mechanical deflection in dental ceramic following adhesive cementation procedures to the tooth structure due to volumetric shrinkage of resin cement during polymerization.

## 2. Materials and Methods

### 2.1. Specimen Preparation

From a solid acrylic resin rod (12.08 ± 0.2 mm in diameter), discs with a thickness of 1.16 ± 0.07 mm were cut with a saw machine (IsoMet Low Speed Saw, Buehler, Lake Bluff, Il USA) and used to produce, by means of the hot-press technique, ceramic discs (*n* = 10) from IPS e.max® Press glass-ceramic material (Lot K16275, low translucency (LT) shade A2, Ivoclar-Vivadent AG, Schaan, Liechtenstein). Four acrylic discs at a time were attached to the sprue base with a 3 mm wax rod and invested with phosphate investment powder (IPS Press Vest lot no. KL2008, Ivoclar-Vivadent AG, Schaan, Liechtenstein) mixed with the special liquid (IPS Press Vest investment liquid lot KL2059, Ivoclar-Vivadent AG, Schaan, Liechtenstein) and distilled water according to the proportion and mixing instruction provided by the manufacturer. The investment ring was filled with the slurry under vibration and left on the bench to set for 60 min. Then, the sprue base was carefully removed and the refractory mould was placed in a burn-out furnace (IPCO 3300, Metrotherm, R GMbH Lilienthal Germany), to eliminate the acrylic. It was heated from room temperature to 250°C at 5°C/min and heat soaked for 30 min. Then, the temperature was increased to 850°C at 9°C/min and held for 60 min. At the end, the refractory mould was removed from the furnace; its pressing channel was filled with one large ceramic ingot together with the plunger. The assembly was then placed in a specifically designed pressing furnace (EP 600 Combi, Ivoclar-Vivadent Schaan, Lichtenstein) heated to 920°C at 60°C/min under vacuum and heat soaked for 25 min, and then the ceramic ingot was automatically pressed with the plunger into the mould at 5 bar pressure. Following the pressing procedure, the assembly was removed from the furnace and allowed to cool to room temperature.

The ceramic discs were carefully divested by means of air-particle abrasion using 90 *μ*m glass beads (Dentalfarm Turin, Italy) at 4 bar pressures until the specimens were visible. Then, the air pressure was reduced to 2 bars and the ceramic discs were air-particle abraded on both sides at the distance of 1 cm to prevent surface damage. The ceramic discs were then ultrasonically cleaned for 15 min in a bath of <1% hydrofluoric acid (Invex liquid, lot K06662 Ivoclar-Vivadent Schaan, Lichtenstein) to remove the reaction layer formed during the heat-pressing procedure. Subsequently, the discs were wet cut, from the sprues under cooling water, at low speed and low-hand pressure, by means of a diamond cutting disc, and the edge was reshaped with a conical diamond bur. Then, the ceramic discs were subjected to air-particle abrasion, on both sides, with 100 *μ*m aluminium oxide (Dentalfarm Turin, Italy) at 1 bar pressure and at a distance of 1 cm, following 15 min of ultrasonic bath in the Invex liquid to completely remove the reaction layer.

A grinding machine (Alpha and Beta Grinder-Polisher, Buehler, USA) with ascending silicon carbide papers of 340, 600, 800, 1200, and 2500 grits (Sic grinding paper Buehler, USA) at a speed of 100 rpm with a load of 12 N was used to reduce, under cooling water, the thickness of the specimens and to polish one surface of the ceramic discs. The final thickness of the specimens, measured with a micrometer accurate to 10 *μ*m (Digimatic Micrometer, Mitutoyo Corp., Tokyo, Japan), was 0.57 ± 0.05 mm. The polished surface of the IPS e.max® Press glass-ceramic discs was labelled as the reference surface, and two points opposite to each other on the edge the specimens were marked to ensure repeating profilometric measurements of the selected area.

### 2.2. Deflection Test

3D profilometric measurements, to characterize the mean of maximum mechanical deflection (*μ*m), were performed on the polished surface of the ceramic disc. To produce accurate measurements, the ceramic discs were placed and held during the duration of the test, in a custom-made levelling metallic device to remove any difference in slope from their surface. Data were recorded across a 10 mm^2^ area (10 mm length and 1 mm width) and for its entire diameter with a contact 90° conisphere diamond stylus (Talysurf CLI 2000, Taylor Hobson Ltd., Leicester, UK) tip of 2 *μ*m radius. The velocity of the contact stylus and its applied load on the surface of the specimen was 1 mm/s and 0.75 mN, respectively. The deflection of the ceramic discs was determined by averaging 250 traces measuring 4 *μ*m step size (*y*-direction) with data points recorded every 10 *μ*m (*x*-direction) at 40 nm resolution (*z*-direction) [16, 17, 19, 20]. Subsequently, to baseline profilometric evaluation of the polished surface, the ceramic discs were annealed in a porcelain furnace (Vita Vacumat 40T Vita Zahnfabrik, Bad Säckingen, Germany) to relieve any residual stress that could be induced during grinding and polishing procedures of the specimens [21]. The ceramic discs were placed with the polished surface in contact with the nitride refractory tray, heated from 200°C to 450°C (below the glass transition temperature (455°C) of IPS e.max® Press glass-ceramic material) in air at 20°C/min, heat soaked for 40 min, and cooled to 60°C at 2.9°C/min.

Following the quantification of the mean of maximum deflection of the annealed specimens, the unpolished surface (nonreference) of the ceramic discs was subjected to air-particle abrasion with 50 *μ*m aluminium oxide (Dentalfarm Turin, Italy) at 2 bar pressures, at the distance of 1 cm and for 10 sec. Then, the polished surface of the ceramic discs was reprofiled to quantify the mean of maximum deflection.

To estimate the magnitude of deflection of the ceramic discs after cement coating, a quantity of 0.025 g of Rely-X™ Veneer Cement (Lot no. 8EB, shade A3, 3M ESPE, St. Paul, MN, USA) was applied to the alumina air-particle-abraded surface of the specimens. The cement was covered with Mylar, and a glass slide was used to press the resin until it spreaded to the edge of the ceramic disc. Then, the cement was light irradiated (Optilux 501, SDS Kerr, Danbury, CT, USA) for 20 sec at the light intensity of 526 mW·cm^−2^, delivered with a 13 mm tip diameter placed in contact with the resin cement. The mean thickness of the resin cement was 93 ± 22 *μ*m. Profilometric measurements were performed subsequent to resin cement coating (0 hr) and after 24, 48, and 168 hrs resin cement coating.

### 2.3. Statistical Analysis

The nonparametric Friedman test followed by Dunn's multiple comparison test was employed to identify significant differences (*p* < 0.05) in maximum deflection of the ceramic discs from polishing (baseline deflection measurement) to annealing alumina particle abrasion of the unpolished surface and at 0, 24, 48, and 168 hrs after cement coating. To perform a comparison between the two different materials, IPS e.max Press and Vitadur Alpha, Student's *t*-test for unpaired data was performed for the three different variables (thickness of discs, thickness of resin cement, and mean of maximum deflection after cement coating at 0 hr).

## 3. Results

**Figure 1:**
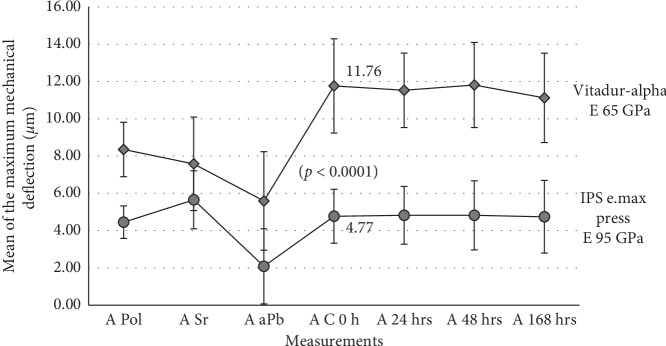
The graph shows the mean of maximum mechanical deflection of the two dental ceramics (IPS e.max® Press glass-ceramic and Vitadur Alpha^*∗*^) characterized by profilometric measurements after polishing (A Pol), after stress relief (A Sr), after alumina air-particle abrasion (A aPb), and after resin cement coating at 0 hr, 24 hrs, 48 hrs, and 168 hrs (A C 0 hr, A 24 hrs, A 48 hrs, and A 168 hrs, respectively). ^*∗*^(data from [16]).

The polished surface of the 10 IPS e.max® Press glass-ceramic discs was characterized by profilometric measurements to have a convex form with a calculated mean of the maximum deflection value of 4.45 ± 0.87 *μ*m (baseline measurements) (Table 1; Figure 1).

**Figure 2:**
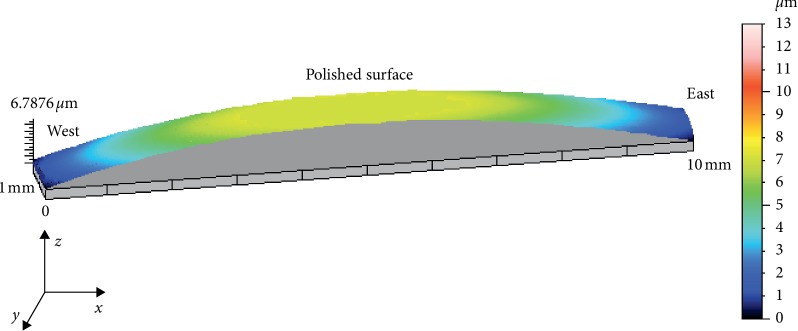
A schematic representation generated by profilometric measurements of an IPS e.max® Press glass-ceramic disc following resin cement coating of alumina air-particle-abraded surface.

Profilometric measurements following annealing treatment of the specimens revealed an increase (not significant) in convexity of the polished surface of the discs with a mean of maximum deflection value of 5.65 ± 1.56 *μ*m. Following alumina air-particle abrasion treatment of the unpolished surface of the ceramic discs, the Friedman test, followed by Dunn's multiple comparison test, highlighted a significant (*p* < 0.0001) reduction in convexity of the polished surface of the annealed specimens (Figure 1). Coating the alumina air-particle-abraded surface of the ceramic discs with resin cement significantly increased (*p* < 0.05) the mean of the maximum mechanical deflection of the polished surface of the specimens when compared with the mean of the maximum mechanical deflection following alumina air-particle abrasion treatment (Figure 2).

The characterized mean of the maximum mechanical deflection of the resin cement-coated specimens did not change over the time investigated.

Student's *t*-test for unpaired data showed a significant difference (*p* < 0.0001) in the mean of maximum mechanical deflection after cement coating at 0 hr between IPS e.max Press and Vitadur Alpha materials (Figure 1Table 2).

## 4. Discussion

Profilometric measurements, following the fabrication of the IPS e.max® Press glass-ceramic discs and polishing procedure, identified a convex form across the polished surface of the specimens, indicating a state of tensile stress (Figure 2). It has been reported that the induced thermal stress is associated to the sintering technique owing to fast cooling of the ceramic discs from the sintering temperature to room temperature [14]. In a recent study for as-fired ceramic discs, Isgrò and the coauthors [17] identified, by means of profilometric measurements, transient tensile stress displayed by a convex form across the surface of the specimen that was in contact with the refractory tray during fast cooling. Transient stress is the result of temperature gradients within the ceramic body, as the surface of the disc in contact with air, from the sintering temperature to room temperature, would cool faster than the surface of the disc that is in contact with the refractory tray [22, 23]. The IPS e.max® Press glass-ceramic discs were fabricated by means of the heat-pressing technique, which in contrary to the sintering technique minimizes induced thermal stress as the ceramic discs were subjected to slow cooling inside the refractory mould. As a result, profilometric measurements of the IPS e.max® Press glass-ceramic discs following the heat-press technique and polishing procedure (baseline measurements) characterized a reduction in the mean of maximum mechanical deflection (4.45 ± 0.87 *μ*m) when compared with the mean of maximum mechanical deflection (8.4 ± 1.5 *μ*m) quantified for Vitadur Alpha [16] subsequent to sintering and polishing procedure. To clean the surfaces of the ceramic discs from the refractory material and reaction layer, it was required a jet of glass beads (90 *μ*m) followed by a jet of alumina particles (100 *μ*m) both driven by compressed air (at 2 and 1 bar pressure respectively) and subsequent ultrasonic cleaning for 15 min in a bath of hydrofluoric acid (<1%). Assuming that the cleaning procedures had the same effect on both surfaces of each ceramic discs, the calculated baseline mean of the maximum deflection value could be the result of the polishing procedure necessarily to polish the surface of the specimens for accurate profilometric measurements.

Annealing the IPS e.max® Press glass-ceramic discs with the polished surface in contact with the refractory tray and below the glass transition temperature (450°C), it was expected a reduction in convexity of the polished surface of the specimens as a result of stress relaxation. However, profilometric measurements identified an increase in convexity of the polished surface (Figure 1), suggesting that a change in microstructure of the ceramic material has occurred. Even though no crystallographic study was done on the microstructure of the ceramic tested, it was assumed that during the heat treatment, a second-phase crystallization has occurred resulting in a dimensional change of the specimen tested [21].

Alumina air-particle abrasion treatment of the unpolished surface of the IPS e.max® Press discs significantly decreased the convexity of the polished surface of the annealed specimens with a characterized mean of the maximum mechanical deflection value of 2.08 ± 2.01 *μ*m (Table 1Figure 1). Alumina air-particle abrasion treatment of the fit surface of a dental ceramic is a routine precementation procedure applied to increase the surface roughness of the ceramic for a better mechanical bonding between the restoration and the resin cement [24]. However, in this investigation, the jet of alumina particles (50 *μ*m) driven by compressed air (at 2 bars at the distance of 1 cm for 10 sec) induced tensile stresses on the unpolished surface of the IPS e.max® Press glass-ceramic discs as well as surface damage [25]. Therefore, the mechanical deformation of the ceramic discs and consequently reduction in convexity of the polished surface of the specimens are attributed to stress relaxation secondary to crack growth, promoted by tensile stress. This finding agrees with the result obtained previously from the study for Vitadur Alpha [16].

By coating with resin cement, the alumina air-particle-abraded surface of the specimens significantly increased the mean of maximum mechanical deflection of the polished surface of the ceramic discs when compared with the mean of maximum mechanical deflection following alumina air-particle abrasion of the unpolished surface. The maximum mechanical deflection of the 10 IPS e.max® Press discs ranged from 2.10 *μ*m to 6.78 *μ*m with a mean value of 4.77 ± 1.44 *μ*m (Figure 1).

During the cement coating procedure, the resin cement infiltrate ceramic surface defects forming a hybrid layer between the ceramic and resin cement [26]. The volumetric shrinkage, within the hybrid layer of the resin cement during polymerization, generated compression stress that induced mechanical deformation throughout the thickness of the IPS e.max® Press glass-ceramic discs. The same behaviour has been observed by the author in the study for Vitadur Apha [16].

Although a previous investigation for resin cement coating IPS e.max® Press glass-ceramic discs (mean thickness of 0.61 ± 0.05) reported a mean BFS value of 288 ± 29 MPa [19] which is not in agreement with the BFS quoted by the manufacture (440 ± 55 MPa) of uncoated discs [13], in contrary, a study for resin cement coating Vitadur Alpha ceramic discs showed a significant increase in the BFS mean value, when compared with the uncoated specimens [9]. The finding of the study for Vitadur Alpha was in agreement with the resin cement strengthening mechanism theory [9, 10].

In the dental literature, it has been reported that the strength and stiffness (modulus of elasticity) of a ceramic progressively increase as the amount of crystal phase in the glass matrix is also increased [4, 27, 28]. In order to relate mechanical deformation of the specimens as a result of resin cement coating procedure with the stiffness property of dental ceramics, the authors have selected the IPS e.max® Press material that has an elastic modulus of 95 GPa [13] to enable comparisons with the study for a Vitadur Alpha [16] material with an elastic modulus of 65 GPa (Figure 1Table 2). In the experimental design of the current investigation, the IPS e.max® Press specimens were treated, following fabrication procedure, in the same way as for Vitadur Alpha specimens [16]. Additionally, the IPS e.max® Press discs had a mean thickness of 0.57 ± 0.05 mm, and the mean thickness of the resin cement was 93.0 ± 22 *μ*m, whereas the mean thickness of Vitadur Alpha ceramic discs and the mean thickness of the resin cement were 0.58 ± 0.03 mm and 103.2 ± 23 *μ*m, respectively. These values were not significantly different. Both IPS e.max® Press glass-ceramic discs and Vitadur Alpha ceramic discs were coated with Rely-X™ Veneer Cement (3M ESPE, St. Paul, MN, USA). The mean of maximum mechanical deflection following resin cement coating of the ceramic discs reported for Vitadur Alpha and that obtained for IPS e.max® Press were 11.8 ± 2.5 and 4.77 ± 1.44 *μ*m, respectively, were significantly different (*p* < 0.0001) (Figure 1Table 2). From the comparison of the two studies, it is suggested that IPS e.max® Press glass-ceramic material that has an elastic modulus of 95 GPa (stiffer compared with Vitadur Alpha (65 GPa)) was more capable to withstand the forces generated by compressive stress as a result of polymerisation shrinkage of the resin cement reducing the mechanical deformation across the thickness of a ceramic discs.

The clinical implication of this study was that the precementation technique namely alumina air-particle abrasion treatment of the fit surface of the ceramic discs and cementation procedure to bond the restoration to the underlying tooth structure have the potential to induce mechanical deformation throughout the thickness of the structure. However, by selecting a dental ceramic material with higher elastic modulus and therefore stiffer and processed with the heat-pressing technique, it would minimize stress-inducing mechanical deformation during fabrication and cementation procedures of a PLV or DBC dental restoration.

## 5. Conclusions

Within the limits of the current study, it may be concluded that

- The deflection test, based on profilometric measurements, is a reliable method that can be employed to characterize stress-inducing mechanical deformation in ceramic discs before crack formation.
- The baseline profilometric measurements, following fabrication of the ceramic discs by means of the heat-press technique and polishing procedure of one surface of the ceramic discs, identified a reduction in convexity when compared with the sintering technique. Annealing treatment was not effective to release stress. However, an increase in convexity of the polished surface was observed, suggesting a change in microstructure as a result of heat treatment. Alumina air-particle abrasion of the unpolished surface of the ceramic discs introduced tensile stress characterized by a significant reduction in convexity of the polished surface of the specimens. Resin cement coating procedure generated compression stress that induced mechanical deformation.
- A ceramic material with high elastic modulus (stiffer) is capable to minimize stress-inducing mechanical deformation during precementation and cementation procedures of a dental ceramic restoration.

## Figures and Tables

**Table 1:** The mean of the maximum mechanical deflection values (*μ*m) and associated standard deviations for IPS e.max® Press glass-ceramic discs measured after polishing (baseline measurements), after stress relief, after alumina air-particle abrasion, and after resin cement coating at 0, 24, 48, and 168 hrs.

Measurements	Mean of the maximum mechanical deflection (*μ*m ± SD)
After polishing	4.45 ± 0.87
After stress relief	5.65 ± 1.56
After alumina air-particle abrasion	2.08 ± 2.01
After resin cement coating at 0 hr	4.77 ± 1.44
After resin cement coating at 24 hrs	4.82 ± 1.55
After resin cement coating at 48 hrs	4.82 ± 1.86
After resin cement coating at 168 hrs	4.74 ± 1.95

**Table 2:** Comparison of the data obtained from the two studies.

Materials	*E* (GPa)	Mean thickness (mm ± SD) of the discs (*N* = 10)	Mean thickness (*μ*m ± SD) of the resin cement	Mean of maximum mechanical deflection (*μ*m ± SD) after cement coating at 0 hr
IPS e.max® Press	95	0.57 ± 0.05	93.0 ± 22	4.77 ± 1.44
Vitadur Alpha^*∗*^	65	0.58 ± 0.03	103.2 ± 23	11.8 ± 2.5

^*∗*^Data from [16]

## Data Availability

The data used to support the findings of this study are included within the article, and necessary explanations in relation to this can be obtained from the corresponding author upon request.
